# Diet-induced Ketoacidosis in a Non-diabetic: A Case Report

**DOI:** 10.5811/cpcem.2020.2.44736

**Published:** 2020-04-23

**Authors:** Sam Slade, John Ashurst

**Affiliations:** Midwestern University, Kingman Regional Medical Center, Department of Emergency Medicine, Kingman, Arizona

**Keywords:** Anion-Gap Metabolic Acidosis, Ketosis, Diet-Induced Ketosis

## Abstract

**Introduction:**

Anion gap metabolic acidosis is a common disorder seen in the emergency department. The differential can include toxicological, renal, endocrine, infectious, and cardiogenic disorders. Ketosis, however, is one of the rarer causes of metabolic acidosis seen by the emergency physician in developed nations.

**Case Report:**

A 53-year-old female presented after starting a low-carbohydrate ketogenic diet for weight loss. She reported xerostomia, nausea with abdominal pain and a 17-pound weight loss over the previous 22 days. Labs revealed an anion-gap metabolic acidosis with ketosis. She was treated with 5% dextrose in normal saline and a sliding scale insulin coverage. Her anion gap corrected during her hospital course and was discharged on hospital day three.

**Discussion:**

The ketogenic diet typically consists of a high-fat, adequate protein and low carbohydrate diet that has previously been thought to be relatively safe for weight loss. However, when carbohydrates are completely removed from the diet an overproduction of ketones bodies results in ketoacidosis. Treatment should be aimed at halting the ketogenic process and patient education.

**Conclusion:**

Although rarely included in the differential for metabolic acidosis, diet-induced ketosis should be included by the emergency physician when faced with a patient who recently changed their eating patterns.

## INTRODUCTION

Anion gap metabolic acidosis is a common, life-threatening diagnosis in the emergency department (ED) with various potential etiologies. CAT MUD PILES is a mnemonic often used to help remember the most common causes of anion gap metabolic acidosis (Table 1). However, starvation ketosis is one of the rarer causes of ketoacidosis. Ketosis is typically prevented with diets that consist of 100 grams (g) of carbohydrates per day, and as little as 7.5 g of glucose a day can decrease ketone production. We present a case of a 53-year-old female with a high anion gap metabolic acidosis whose history and workup did not correlate with traditional causes. She was found to have a ketoacidosis caused by a zero carbohydrate diet used for weight loss. It is important to raise awareness of this disease, as the popularity of protein- and fat-rich diets that minimize carbohydrate intake are becoming increasingly popular.

## CASE REPORT

A 53-year-old female presented to the ED with six days of nausea and vomiting. She also noted feeling progressively weak with xerostomia and lower abdominal pain for several days prior to presentation. Further history revealed that she was a previous vegetarian who over the prior 22 days was attempting to lose weight by eating solely meat and eggs. She reported a 17-pound weight loss over the time period and noted that she was consuming only minimal carbohydrates. She took no medications and denied chronic alcohol use.

Her vitals upon arrival were as follows: temperature 97.1 degrees Fahrenheit, heart rate 77 beats per minute, respiratory rate 16 breaths per minute, blood pressure 160/106 millimeters of mercury, and oxygen saturation 97% on room air. She weighed 73 kilograms with her stated height of 5 feet 6 inches and had a body mass index of 26. The physical exam revealed dry mucous membranes and a benign abdominal exam.

The patient was given two liters (L) of normal saline intravenously (IV) and four milligrams of ondansetron IV for nausea. Labs were drawn due to a concern for abnormal electrolytes as well as her change in diet. Labs revealed an anion gap acidosis with ketosis (Table 2).

In the ED she received antiemetics, two L of normal saline and an infusion of D5NS at 150 cubic centimeters (cc) an hour, and her symptoms greatly improved. By the time she was admitted to the hospital, her gap had closed to a level of 12. She continued to receive an infusion of 5% dextrose in normal saline (D5NS) at 150 cc an hour as well as insulin subcutaneously on a sliding scale after admittance to the hospital. Her anion gap remained within the normal limits while in the hospital and her insulin was discontinued. After returning to a normal diet, her glucose remained stable and she was discharged after three days with only potassium supplement prescriptions. Since discharge, she established a balanced diet and symptoms ceased.

**CPC-EM Capsule**

What do we already know about this clinical entity?

The ketogenic diet consists of a high-fat, adequate protein and low carbohydrates. When carbohydrates are removed however, ketone bodies can form, causing ketoacidosis.

What makes this presentation of disease reportable?

Although ketoacidosis is common with other disorders, diet-induced ketoacidosis is typically not included in the differential of most emergency physicians (EP).

What is the major learning point?

Diet-induced ketoacidosis should be included in patients with an unexplained metabolic acidosis and further history should be gathered into any recent dietary changes.

How might this improve emergency medicine practice?

The EDP should keep a broad differential when faced with a patient with metabolic acidosis and search for the underlying cause.

## DISCUSSION

The ketogenic diet is a high-fat, adequate protein, low-carbohydrate diet that could be beneficial for diseases such as epilepsy, but the emergency provider is most likely to see its use during episodes of weight loss.^1^ After only several days of low-carbohydrate dieting, the body depletes its glucose stores and adipose cells begin ketogenesis to supply the brain with glucose.^1^ During this process, there is an overproduction of acetyl-CoA, which produces beta-hydroxybutyrate, acetoacetate, and acetone in the liver.^1^ These ketone bodies are then used as a source of energy for the body, but their overproduction could lead to ketoacidosis.^1^

Diet-induced ketosis is more likely to occur in children, and pregnant or lactating females due to lower glycogen stores, inherent insulin resistance, and increased lipolysis from pregnant and lactating females.^2^ While starvation ketosis usually does not result in a bicarbonate level less than 18 milliequivalents per liter (mEq/L),^3^ our patient’s initial bicarbonate level was 8 mEq/L. Based upon the patient’s medical history and risk factors, she should not have reached the stage of ketoacidosis. However, the lack of carbohydrates in her diet increased the possibility of a component of starvation ketosis.

Ketogenic diets have been proven to be safe and effective in treating obesity and have shown that patients do not develop anion gap acidosis due to the diet.^4^ A similar case related to the Atkins diet was reported in 2004 and had a similar presentation.^5^ There have been at least four more cases of low-carb, high-protein diets causing high anion gap metabolic acidosis with associated ketones in the blood or urine.^6^ All cases involved women and had a similar positive outcome.

Treatment should be aimed at halting the ketogenic process through the consumption of carbohydrates or providing the patient with intravenous fluids that contain dextrose. Patients with any underlying conditions that may cause them to be prone to ketoacidosis should avoid a ketogenic diet regimen. These conditions would include, but are not limited to, chronic alcoholism, pregnancy, lactation and diabetes. In addition, any person on the ketogenic diet should eat at least 100 g of carbohydrates a day to avoid ketoacidosis.

## CONCLUSION

Diet-induced ketoacidosis is a rare disease and may be difficult to diagnose due to incomplete diet history and similarity to other common diseases. To avoid this, the patient should be asked about diet history especially in those who present with vomiting, diarrhea, and symptoms similar to diabetic ketoacidosis without a history of diabetes. Prevention is imperative with this disease and can be avoided by eating a minimum amount of carbohydrates daily. Also patients who are chronic alcoholics, pregnant, lactating, or diabetic should not participate in this diet due to a high risk of ketoacidosis.

Reference values are affected by many variables, including the patient population and laboratory methods used. The range was comprised from Kingman Regional Medical Center adults who were not pregnant and did not have medical conditions that could affect the results. Therefore, they may not be appropriate for all patients.

## Figures and Tables

**Table 1:** Common causes of metabolic acidosis presented as a mnemonic CAT MUD PILES.

C
carbon monoxide
cyanide
congenital heart failure
A
aminoglycosides
T
Theophylline
Toluene
M
Methanol
U
Uremia
D
Diabetic Ketoacidosis
Alcoholic Ketoacidosis
Starvation Ketoacidosis
P
Paracetamol/acetaminophen
Phenformin
Paraldehyde
I
Iron
Isoniazid
Inborn errors of metabolism
L
Lactic acidosis
E
Ethanol
Ethylene glycol
S
Salicylates

**Table 2:** Laboratory data.

Variable	Reference range, adults	On presentation to the emergency department	On admission to the hospital	Hospital day 2	Hospital day 3/discharge
Sodium (mEq/L)	137–145	139	141	139	139
Potassium (mEq/L)	3.5–5.1	4.8	3.8	2.8	2.8
Chloride (mEq/L)	100–1 08	103	111	105	97
Carbon dioxide (mEq/L)	22–30	8	18	26	34
Glucose (mg/dL)	74–1 06	163	179	159	102
Urea nitrogen(mg/dL)	6–20	12	10	6	6
Creatinine (mg/dL)	0.52–1.04	0.70	0.60	0.50	0.50
Calcium (mg/dL)	8.4–10.2	10.2	9.1	9.0	8.8
Phosphorus (mg/dL)	2.5–4.5	N/A	2.1	1.9	2.8
Magnesium (mg/dL)	1.6–2.3	N/A	2.0	1.9	1.9
Anion gap	1–12	28	12	8	8
Osmolality, calculated (mOs/kg)	225–285	281	285	279	275
Osmolality, serum	275–295	302	N/A	N/A	N/A
Albumin (g/dL)	3.5–5.0	5.3	N/A	N/A	N/A
Alcohol, (ethanol) (mg/dL)	0–10	< 10	N/A	N/A	N/A
Acetaminophen (ug/mL)	10–30	<10	N/A	N/A	N/A
Salicylate (mg/dL)	0–2	<1	N/A	N/A	N/A
Acetone (mmol/L)	0.0–.06	4.9	3.4	N/A	N/A
Lactic acid (mmol/L)	0.7–2.0	1.4	N/A	N/A	N/A
Urinalysis					
Leukocyte esterase	Negative	Negative	N/A	N/A	N/A
Nitrites	Negative	Negative	N/A	N/A	N/A
Protein (mg/dL)	Negative	100	N/A	N/A	N/A
Glucose (mg/dL)	Negative	50	N/A	N/A	N/A
Ketones (mg/dL)	Negative	80	N/A	N/A	N/A
Specific gravity	1.003–1.035	1.024	N/A	N/A	N/A
Arterial blood gas					
pH	7.35–7.45	7.289	N/A	N/A	N/A
pCO_2_ (mm Hg)	35.0–45.0	23.7	N/A	N/A	N/A
pO_2_ (mm Hg)	80.0–100.0	93.2	N/A	N/A	N/A
Bicarbonate (mEq/L)	22.0–26.0	11.1	N/A	N/A	N/A
Hemoglobin A1c (%)	4.0–5.6	5.8	N/A	N/A	N/A

*mEQ*, milliequivalent; *L*, liter; *mg*, milligram; *dL*, deciliter; *N/A*, not available; *mOs*, milliosmoles; *kg*, kilogram; *g*, grams; *ug*, micrograms; *mL*, milliliter; *mmol*, millimole; *pCO_2_*, partial pressure of carbon dioxide; *mm Hg*, millimeters of mercury; *pO_2_*, partial pressure of oxygen.
